# Expression and prognostic potential of ribosome 18S RNA m^6^A methyltransferase METTL5 in gastric cancer

**DOI:** 10.1186/s12935-021-02274-3

**Published:** 2021-10-26

**Authors:** Zhenshuang Wang, Jingwei Liu, Yi Yang, Chenzhong Xing, Jingjing Jing, Yuan Yuan

**Affiliations:** 1 Tumor Etiology and Screening Department of Cancer Institute and General Surgery, The First Hospital of China Medical University, Shenyang, 110001 China; 2 Key Laboratory of Cancer Etiology and Prevention in Liaoning Education Department, the First hospital of China Medical University, Shenyang, 110001 China; 3 Key Laboratory of Gastrointestinal Cancer Etiology and Prevention in Liaoning Province, the First Hospital of China Medical University, Shenyang, 110001 Liaoning Province China; 4 Department of Neurosurgery of the First Hospital of China Medical University, Shenyang, 110001 China

**Keywords:** METTL5, Gastric cancer, Prognostic biomarkers, Nomogram

## Abstract

**Background:**

Ribosomal RNA N6-methyltransferase METTL5 was reported to catalyze m^6^A in 18S rRNA. We aimed to investigate the expression and prognostic features of METTL5 in gastric cancer (GC).

**Methods:**

In this study, 168 GC patients and their corresponding adjacent tissues were collected. Immunohistochemical staining was used to detect the expression of METTL5 protein. Univariate and multivariate Cox analysis were used to dertermine the prognostic role of METTL5 protein in GC, and a nomogram was constructed to evaluate GC patients’ prognosis based on METTL5 expression. Data from TCGA and GEO database were also used to validate the prognostic value of METTL5 in GC patients on mRNA level. We further performed GSEA enrichment analysis to explore the possible function and related pathways related to METTL5.

**Results:**

METTL5 protein in gastric cancer tissues (GCTs) was significantly decreased compared with adjacent normal tissues (ANTs) and adjacent intestinal metaplasia tissues (AIMTs) (P < 0.001, respectively). Meanwhile, METTL5 expression was negatively correlated with clinicopathologic stage. According to multivariate Cox proportional hazards model analysis, METTL5 protein expression was a good independent predictor of GC prognosis (p < 0.05). Patients with high METTL5 expression had better prognosis. The nomogram constructed based on METTL5 expression could predict the prognosis of GC patients well. GSEA analysis showed that genes of METTL5 low expression group were enriched in some oncogenic signaling pathways such as ERBB, MAPK, JAK-STAT, Wnt, and mTOR, as well as some immune pathways, including Fc-gamma R mediated phagocytosis, Fc-epsilon Ri, chemokine, T cell receptor and B cell receptor signaling pathway. While the high expression group of METTL5 was mainly related to oxidative phosphorylation, nucleotide excision repair and mismatch repair.

**Conclusions:**

METTL5 protein was decreased in GCTs compared with AIMTs and ANTs, and it may be a potential prognostic biomarker in GC.

**Supplementary Information:**

The online version contains supplementary material available at 10.1186/s12935-021-02274-3.

## Background

Gastric cancer (GC) is one of the most common malignant tumors with the third highest cancer mortality worldwide [1]. The development and progression of GC is a complicated multistep process, including a plenty of genetic and epigenetic changes [2]. The poor prognosis of GC may be due to a lack of early diagnostic efficiency and incorrect prognosis prediction. Therefore, further researches into the underlying mechanisms of development and progression, and the identification of risk and prognostic biomarkers, are important to improve early detection and effective treatment of GC.

Ribosomal RNA (rRNA) is one of the major ribonucleic acids in living cells and is essential for the production of functional ribosomes, which control protein synthesis and cell function through the translation of the genetic code [3]. Transcription and processing of RNA are very important for cancer cells that need a large number of protein translation to maintain cell growth [3]. RNA enzymes play a crucial part in the processing and maturation of rRNA [4].

M^6^A modification is a kind of reversible RNA modification in which adenosine is added to RNA via a methyltransferase complex and removed by m^6^A demethylase [5]. Recent studies on m^6^A have proved that the dynamic and reversible regulation of RNA modification could regulate important biological processes such as RNA metabolism, processing and directional differentiation of stem cells [6, 7]. M^6^A RNA methylation is modulated by a series of epigenetic modulator enzymes termed as “writers”, “erasers” and “readers”. The expression level of these m^6^A regulatory proteins such as METTL3, FTO, YTHDF2 have been found to be dysregulated in a variety of cancers, including lung cancer, colorectal cancer, breast cancer and GC, etc. [8–11].

METTL5, as a ribosomal RNA N6-methyltransferase, can catalyze m^6^A in human 18S rRNA at position A1832 site, and may regulate the function and development of ribosome [12, 13]. Previous studies have shown that when mouse embryonic stem cells (MESCs) lack METTL5, the cell translation rate is reduced, the pluripotency is spontaneously lost, and the differentiation potential is impaired [14, 15]. Bi-allele variation of METTL5 could lead to autosomal recessive mental retardation and microcephaly and METTL5 was necessary for normal walking in fruit flies [16, 17]. In addition, METTL5 has been reported to promote translation initiation and cell growth in breast cancer. It was also upregulated and associated with poor prognosis in lung adenocarcinoma [12, 18]. Currently, the expression characteristics of METTL5 and its prognostic potential in GC are still unknown.

In our study, the protein expression of METTL5 was detected in gastric cancer tissues (GCTs) and adjacent tissues by immunohistochemical staining. Further, the correlation between the expression level of METTL5 and the clinicopathological parameters of GC patients, as well as the effect of METTL5 protein on GC prognosis were investigated. The study aims to provide novel clues for revealing the expression and prognostic potential of ribosomal RNA N6-methyltransferase METTL5 in GC.

## Methods

### Study design

**Fig. 1:**
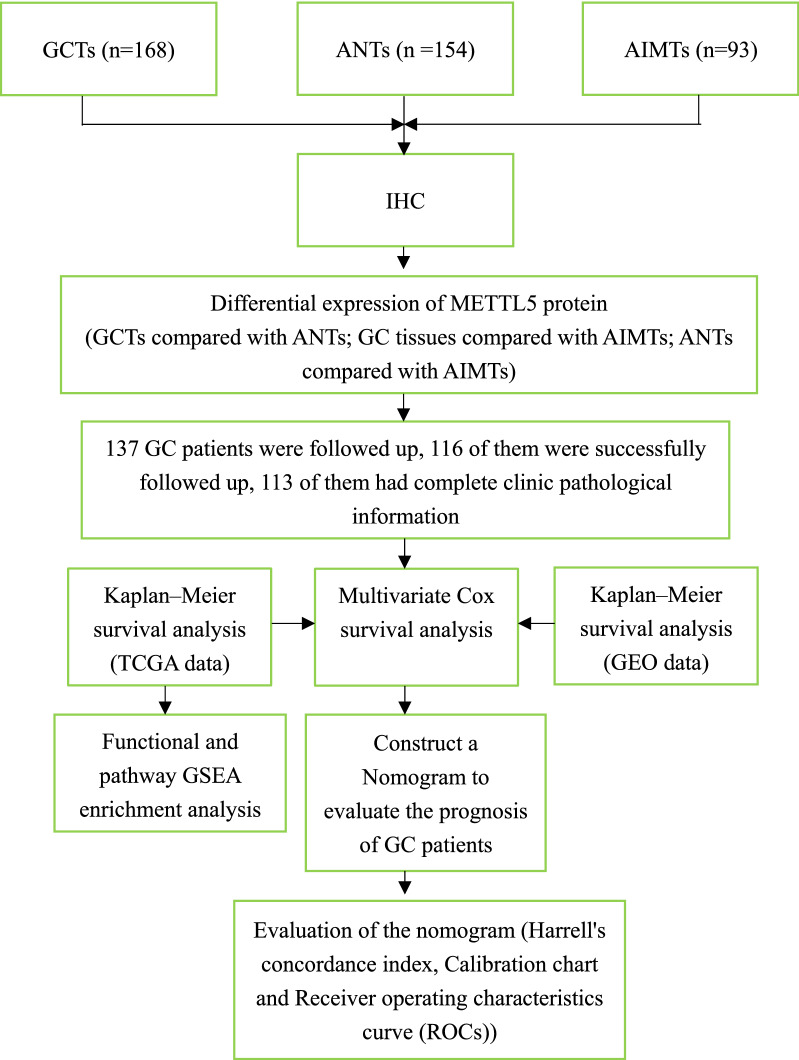
The overall technical roadmap for this article. GCTs: gastric cancer Tissues, ANTs: adjacent normal tissues, AIMTs: adjacent intestinal metaplasia tissues

METTL5 immunohistochemistry was performed on collected GCTs, adjacent normal tissues (ANTs) and adjacent intestinal metaplasia tissues (AIMTs), and then scored. Subsequently, differential expression analysis and survival analysis were performed on this group of samples. Based on the results of follow-up, we constructed and validated the nomogram to evaluate the prognosis of GC patients. Meanwhile, bioinformatics analysis methods and data sets in TCGA and GEO were used to verify the effect of METTL5 expression on the prognosis of GC patients, and GSEA analysis was performed using TCGA data to predict the possible molecular mechanism of METTL5 in GC. The details are described in the following methods. We present the flow chart of the overall study design in Fig. 1.

### Patients and tissues specimens

This study was approved by the Research Medical Ethics Committee of the First Affiliated Hospital of China Medical University, with the written informed consent of the participants. In our study, a total of 168 GCTs, 154 ANTs and 93 AIMTs were obtained from the First Affiliated Hospital of China Medical University from February 2013 to December 2018. International Union Against Cancer (UICC)/American Joint Committee on Cancer (AJCC) (eighth edition, 2018) was applied to determine TNM staging of GC in the following of postoperative pathological diagnosis. We followed up 137 patients with GC, of which 116 patients were successfully followed up. In addition, 3 patients were excluded in the prognostic analysis due to incomplete clinicopathological information. Finally, a total of 113 patients were analyzed for prognosis. Defined overall survival was the period from the date a patient accepted surgery to the date of death. The final follow-up visit was completed by December 2019, and the overall mean follow-up time is 854 days (28.47 months).

### Immunohistochemistry and scoring

Cut 4-μm-thick paraffin-embedded tissue sections into the poly-l-lysine-coated glass slides and wax them in an oven at 65 °C overnight. Tissue sections were deparaffinized with xylene, rehydrated with ethanol, and immersed in EDTA buffer for antigen recovery. The endogenous peroxidase was quenched with 3% hydrogen peroxide for 30 min. Tissue collagen was then blocked with 10% normal goat serum for 30 min to bring down nonspecific staining. Then tissue sections were incubated with rabbit polyclonal anti-METTL5 antibody (dilution 1:1000; NBP1-56,640, Novus Biologicals, Littleton, CO, USA) at a room temperature (25–28 °C) for 60 min. Then, the tissue sections were incubated with bio-acylated secondary antibody and streptavidin–biotin-peroxidase for 15 min, respectively. Tissues were stained with DAB chromogenic reagent for 60 s (DAB-1031, Maixin Inc., Fujian, China), then counterstained with hematoxylin. PBS buffer was used instead of METL5 antibody as a negative control. UltraSensitive™ SP were applied in our study (Mouse/Rabbit) IHC Kit (KIT-9720, Maixin Inc., Fujian, China).

The immunohistochemical results were evaluated by two experienced pathologists. Positive staining intensity score: 0, negative; 1–4, weak positivity ( +); 5–8 medium positivity (+ +), 9–12 strong positivity (+ + +). The positive staining degree was scored according to the percentage of positive cells in each lesion: (0, 0–5; 1, 6–25; 2, 26–50; 3, 51–75; 4, 76–100%. The final score was calculated well and truly by multiplying the positive and intensity scores, obtaining a score of 0 to 12.

### Construction and evaluation of the nomogram

Independent prognostic factors filtered by multivariable Cox proportional hazards models were used to construct a prognosis prediction model of GC, including METTL5 expression level, TNM stage tumor location, differentiation degree and perineural invasion. In this nomogram, a score was defined for each risk factor, and a new risk classification system was established. Base on the median, all the patients were divided into the low-risk group and the high-risk group respectively, according to the patient total risk score in the model. Harrell's concordance index (C-index) and the calibration chart were applied to assess the performance of the prediction model. Receiver operating characteristics (ROCs) curve was applied to assess the precision of the 3- and 5-year survival of the nomogram.

### Kaplan–Meier survival analysis of METTL5 in GC patients

To further investigate the prognostic value of METTL5 in GC patients, we performed Kaplan–Meier survival analysis using METTL5 mRNA expression data of GC from TCGA database. In addition, we also used the online website Kaplan–Meier Plotter ([19] to perform prognostic analysis on multiple GEO datasets of GC, including GSE14210 (n = 145), GSE15459 (n = 200), GSE22377 (n = 43), GSE29272 (n = 268), GSE51105 (n = 94), and GSE62254 (n = 300). The attached table shows the details of the GEO datasets (Additional file 1: Table S1). Survival analysis was performed on mRNA chip/microarray data from 881 GC patients who were divided into high-risk and low-risk groups based on METTL5 median expression.

### Functional and pathway enrichment analysis

We downloaded the data of 375 patients with GC from the UCSC Xena website (https://xena.ucsc.edu/), and divided them into high- and low- expression group based on the median value of METTL5 expression level. GSEA examined the function and pathway of gene enrichment at the top of both groups. For each test, the number of genome lines was set to 1,000. False discovery rate (FDR), the nominal (NOM) P value, and normalized enrichment score (NES) were applied to ascertain the functional and pathways enriched in each phenotype.

### Statistical analysis

SPSS version 22.0 and GraphPad Prism 8 were used to process the data. R software version 4.0.2 GraphPad Prism 8 were used to draw pictures. Adobe Illustrator CS6 software was applied to further process the pictures. P < 0.05 was used as the criteria for statistical analysis of all data.

## Results

### Expression features of METTL5 protein in different gastric lessions

**Fig. 2:**
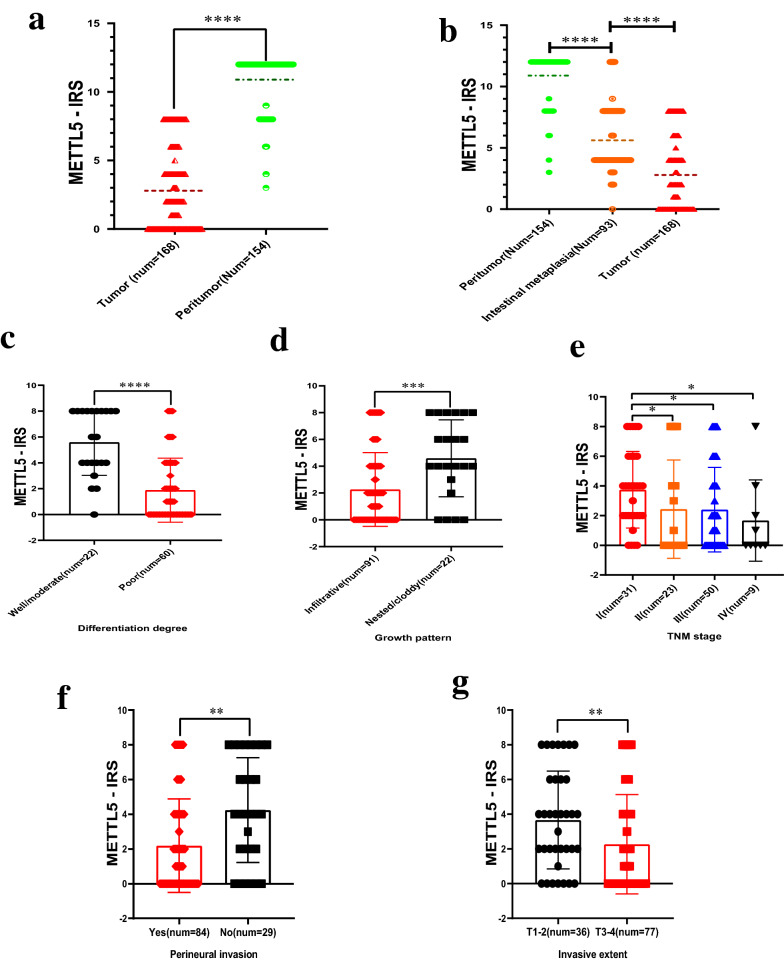
Different expression of METTL5 in different tissues of GC. **a** Differences of METTL5 expression in 168 GCTs and 154 ANTs. **b** Differences of METTL5 expression in 168 GCTs and 154 ANTs and 93 AIMTs. **c**–**f** Differences of METTL5 expression in some subgroup, including differentiation degree, growth pattern, TNM stage, Perineural invasion and invasion extent. (*: P < 0.05; **: P < 0.01; ***: P < 0.001:**** P < 0.0001). IRS, immunoreactivity score

**Fig. 3:**
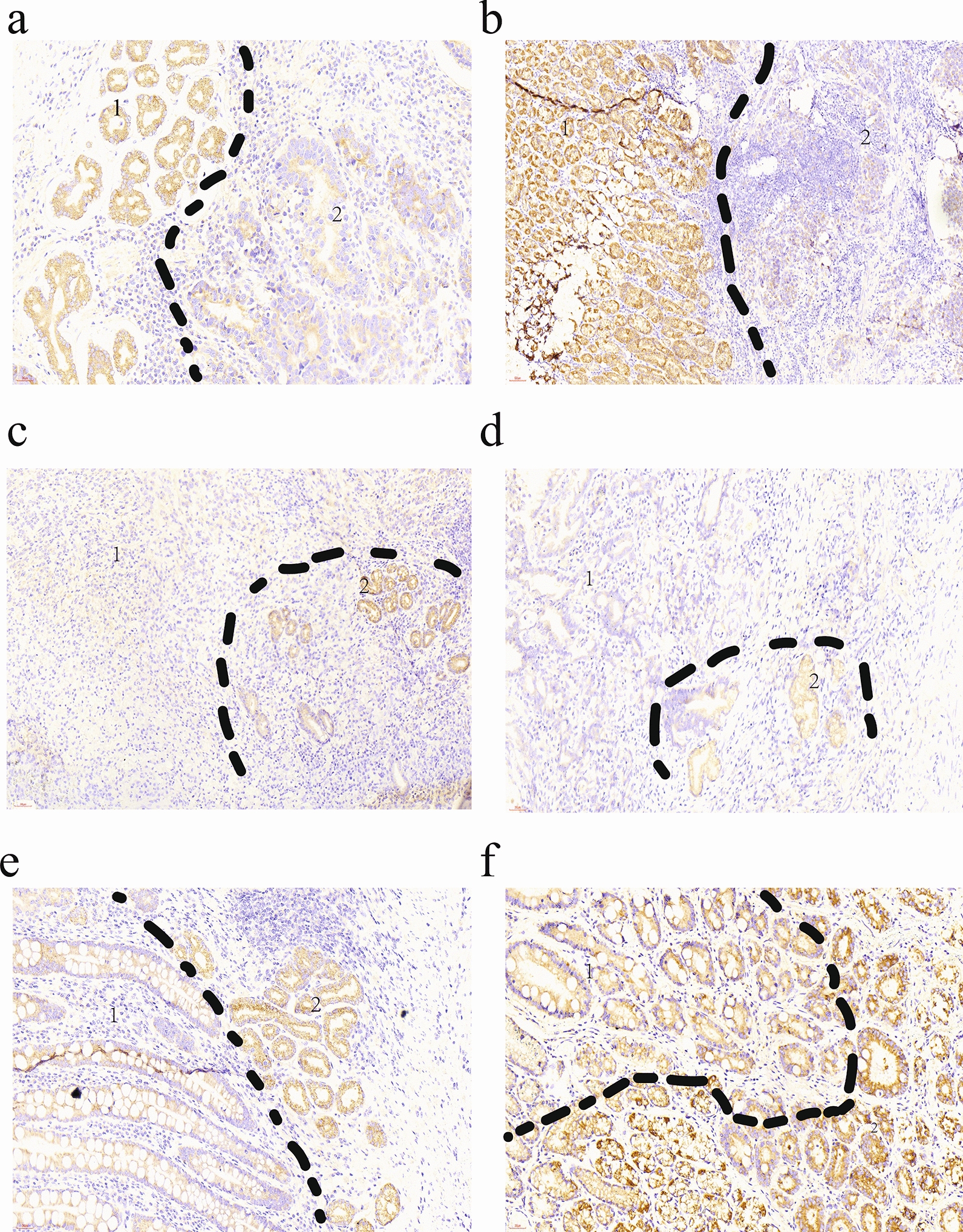
Microscopic images of IDH. **a1**, **b1**, **c2**, **d2**, **e2**, **f2** METTL5 expression in ANTs. **a2**, **d1** METTL5 expression in well differentiated GCTs. **b2**, **c1** METTL5 expression in poor differentiated GCTs. **e1**, **f1** METTL5 expression in AIMTs

In our study, we found that the level of METTL5 protein expression in ANTs (num = 154) was significantly higher than that in GCTs (num = 168) (p < 0.0001) (Fig. 2a). On the same immunohistochemical sheet, almost no staining was observed in GCTs, while strong staining was observed in ANTs (num = 154) (Fig. 3a–d) (Table 1).

**Table 1:** METTL5 expression in gastric cancer tissues, adjacent normal tissues and adjacent intestinal metaplasia tissues

Category	Group	Case (–) n%	Case ( +) n%	Case (+ +) n%	Case (+ + +) n%	PR (%)	p
Overall	GC	65	63	40	0	61.31%	* < 0.0001
	ANT	0	3	33	118	100%	** < 0.0001
	AIM	1	56	28	8	98.92%	*** < 0.0001

PR: positive rate. ANTs: adjacent normal tissues. AIMTs: adjacent intestinal metaplasia tissues. Negative ( −), light positive ( +), positive (+ +), strong positive (+ + +) staining. *: Gastric cancer tissues (GCTs) compared with ANTs, **: GCTs compared with AIMTs, ***: ANTs compared with AIMTs. Mann–Whitney U- test of nonparametric test to analysis

In addition, METTL5 protein expression in ANTs (num = 154) was observed higher than that in AIMTs (num = 93) of the stomach (p < 0.0001), and METTL5 protein expression in AIMTs (num = 93) was also significantly higher than that in GCTs (p < 0.0001) (Fig. 2b, Fig. 3c–f).

Through subgroup analysis, we found that the expression of METTL5 protein was significantly higher in well/moderate differentiated GCTs than in other types, and METTL5 protein was almost not expressed in poorly differentiated GCTs (Figs. 2c, 3a2, c1). The differentially expressed results were also seen in the subgroups of invasive extent depth (T1-2 higher than T3-4) (p < 0.001), TNM stage (II, III, IV lower than I) (p < 0.01), perineural invasion (yes lower than no) (p < 0.001), and growth pattern (infiltrative lower than nested/cloddy) (p < 0.0001). No differences were found in other subgroups (Fig. 2d–g).

### Correlation between METTL5 and clinicopathological parameters in GC patients

By analyzing the correlation between the expression of METTL5 and clinicopathological parameters in 113 GC patients, we found that the expression of METTL5 protein was closely associated with invasive extent, TNM stage, differentiation degree, growth pattern and perineural invasion other than clinical parameters such as distant metastasis, gender, age, lymph node metastasis, tumor location, maximum diameter and lymphatic/venous invasion (Table 2).

**Table 2:** Association between METTL5 and clinicopathological parameters in gastric cancer

Variables	Case (–) n%	Case ( +) n%	Case (+ +) n%	Case (+ + +) n%	PR%	r/ CI95%	p
Gender						− 0.11/[− 0.29, 0.08],	0.22
Male	32	27	16	0	57.33		
Female	11	17	10	0	71.05		
Age						− 0.09/[− 0.27, 0.08]	0.36
≥ 60	20	25	14	0	66.10		
< 60	23	19	12	0	57.41		
Invasive extent						0.26/[0.07, 0.41]	0.01
T1–2	7	18	11	0	80.56		
T3–4	36	26	15	0	53.25		
Lymph node metastasis						0.18/[− 0.01, 0.37]	0.05
Yes	31	22	15	0	54.41		
No	12	22	11	0	73.33		
TNM stage						0.08/[0.02, 0.22]	0.03
I	4	18	9	0	87.10		
II	13	5	5	0	43.48		
III	21	18	11	0	58.00		
IV	5	3	1	0	44.44		
Tumor location						0.02/[0.00, 0.13]	0.64
Gastric body	8	8	7	0	65.22		
Gastric angle	12	6	8	0	53.85		
Antrum of stomach	16	25	9	0	68.00		
Preventriculus	3	5	1	0	66.67		
All	4	0	1	0	20.00		
Maximum diameter (cm)						− 0.03/[− 0.21, 0.13]	0.76
≤ 4	15	19	8	0	64.29		
> 4	28	25	18	0	60.56		
Differentiation degree						0.23/[0.12, 0.41]	< 0.01
Well/moderate	1	9	12	0	95.45		
Mucinous	8	5	5	0	55.56		
Poor	29	23	8	0	51.67		
Others	5	7	1	0	61.54		
Growth pattern						− 0.31/[− 0.50, − 0.12],	< 0.01
Infiltrative	39	36	16	0	57.14		
Nested/cloddy	4	8	10	0	81.82		
Lymphatic/venous invasion						− 0.11/[− 0.28, 0.08]	0.27
Yes	26	23	13	0	58.06		
No	17	21	13	0	66.67		
Perineural invasion						− 0.30/[− 0.45, − 0.11],	< 0.01
Yes	37	33	14	0	55.95		
No	6	11	12	0	79.31		

PR: positive rate. Negative ( −), light positive ( +), positive (+ +), strong positive (+ + +) staining

The association of METTL5 expression with multitaxonomic variables, including TNM stage, Tumor location and differentiation degree, was analyzed by Kruskal–Wallis H- test of nonparametric test. For other dichotomous variables, Mann–Whitney U- test of nonparametric test was used

### Relationship between METTL5 protein expression and GC prognosis

**Fig. 4:**
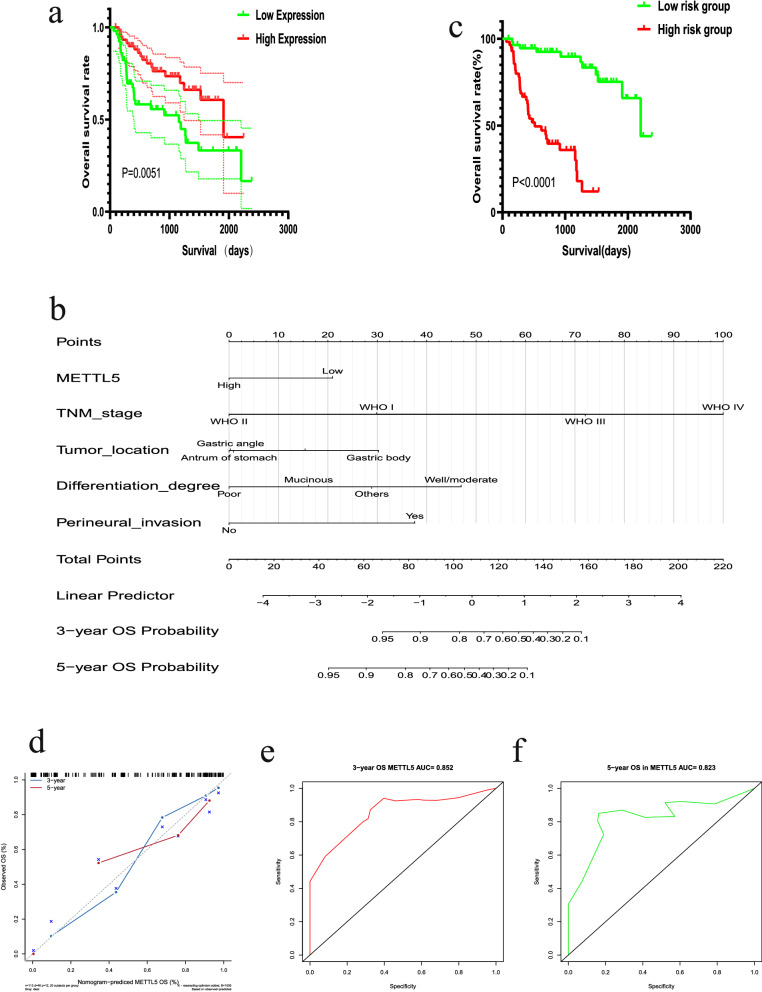
Survival analysis of METTL5, construction and evaluation of the nomogram with METTL5 expression. **a** Poor prognosis of METTL5 expression in GC patient based on Kaplan–Meier survival analysis. **b** Nomogram based on GC data with METTL5 expression and multiple risk factors. **c** Prognostic analysis of high—and low-risk groups based on multiple prognostic risk factors. **d** Calibration curve of nomogram **e–f** Evaluate ROC curves for predicting 3-, and 5-year survival rates in GC patients

METTL5 protein was investigated to indicate the prognosis of GC patients, we divided them into high- and low-expression groups based on the median expression of METTL5 protein, and Kaplan–Meier survival analysis manifested that the prognosis of the group with high METTL5 protein expression was significantly better than that of the group with low METTL5 protein expression (p < 0.001) (Fig. 4a). In addition, according to multivariate Cox proportional hazards model analysis, METTL5 protein was a good independent predictor of GC (p < 0.05) (Table 3).

**Table 3:** Multivariate Cox analysis of gastric cancer

Risk factors	p-value	HR	95% CI	
			The lower limit	The higher limit
Gender	0.33			
Male		Reference		
Female		0.67	0.30	1.50
Age	0.52			
≥ 60		Reference		
< 60		0.79	0.38	1.63
METTL5	** < 0.05**			
High		Reference		
Low		2.28	1.01	5.15
Invasive extent	0.71			
T1–2		Reference		
T3–4		0.58	0.03	10.18
Lymph node metastasis	0.72			
Yes		Reference		
No		0.66	0.07	6.11
TNM stage	** < 0.01**			
I		Reference		
II	0.55	0.40	0.02	8.16
III	0.31	10.20	0.12	
IV	0.11	39.54	0.45	346.25
Tumor location	**0.02**			
Gastric body		Reference		
Gastric angle	< 0.01	0.18	0.06	0.55
Antrum of stomach	< 0.01	0.28	0.11	0.71
Preventriculus	0.27	0.48	0.13	1.75
All	0.12	0.33	0.07	1.37
Maximum diameter (cm)	0.12			
≤ 4		Reference		
> 4		0.54	0.25	1.17
Differentiation degree	**0.01**			
Well/moderate		Reference		
Mucinous	< 0.05	0.26	0.07	1.00
Poor	< 0.01	0.12	0.03	0.47
Others	0.40	0.56	0.15	2.17
Growth pattern	0.69			
Infiltrative		Reference		
Nested/cloddy		1.34	0.32	5.54
Lymphatic/venous invasion	0.72			
Yes		Reference		
No		0.66	0.07	6.11
Perineural invasion	**0.02**			
Yes		Reference		
No		0.14	0.03	0.70

*HR* hazard ratio, *CI* confidence interval

### Construction and evaluation of a METTL5-related prognostic nomogram in GC

According to the consequences of the multivariate Cox regression analysis, the nomogram for overall survival (OS) of GC patients was constructed based on all significant variables, including METTL5 expression level, TNM stage, tumor location, differentiation degree and perineural invasion (Fig. 4b). In this model, a score was defined for each risk factor. Then, according to the total score of each patient, the cutoff value was selected using median, and a risk classification system was established. Moreover, the Kaplan–Meier method and the log-rank test were applied to perform and distinguish the survival outcome of two risk groups (low-risk group and high-risk group). The verdict declared that GC patients (< 105 points) in the low-risk group had longer OS than the high-risk group (> 105 points) (Fig. 4c). Based on the patient's overall score, we can clearly extrapolate each patient's 3-year and 5-year survival and determine their risk for survival.

**Fig. 5:**
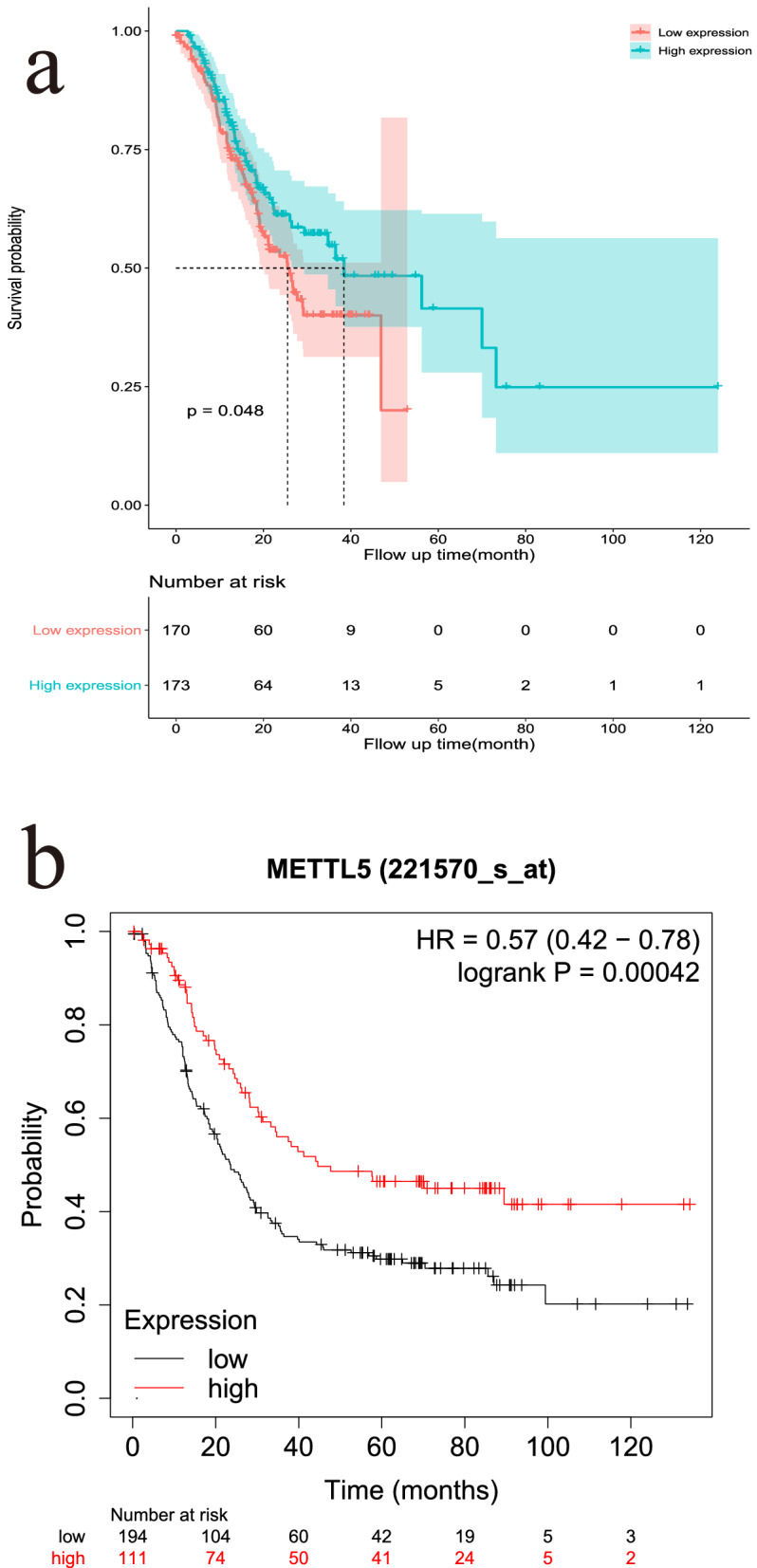
Kaplan–Meier survival analysis of METTL5 in GC patients.** a** Data from overall TCGA database. **b** Data from GEO database (GSE14210 (n = 145), GSE15459 (n = 200), GSE22377 (n = 43), GSE29272 (n = 268), GSE51105 (n = 94), GSE62254 (n = 300))

In the GC patients set, the C-indexes of OS was 0.827(95% CI 0.754–0.899), suggesting that this set of data has good internal consistency. In addition, calibration curves for the nomogram did not deviate from the reference line, which can better predict credibility. (Fig. 4d) In order to confirm whether the nomogram could predict the prognosis of GC patients well, the time-dependent ROC curve of the OS was plotted, and the area under ROC curve (AUC) values of the 3- (Fig. 4e) and 5- (Fig. 4f) year OS rates were 0.852 and 0.823, respectively. In conclusion, the results suggested that our model can well predict the prognosis of GC patients.

### Kaplan–Meier survival analysis of METTL5 in GC patients

According to the analysis results of data from the TCGA and GEO datasets, GC patients with low METTL5 expression had poor prognosis (p < 0.05). This finding was consistent with our experimental results (Fig. 5a, b).

### Functional and pathway enrichment analysis of METTL5 in GC

**Fig. 6:**
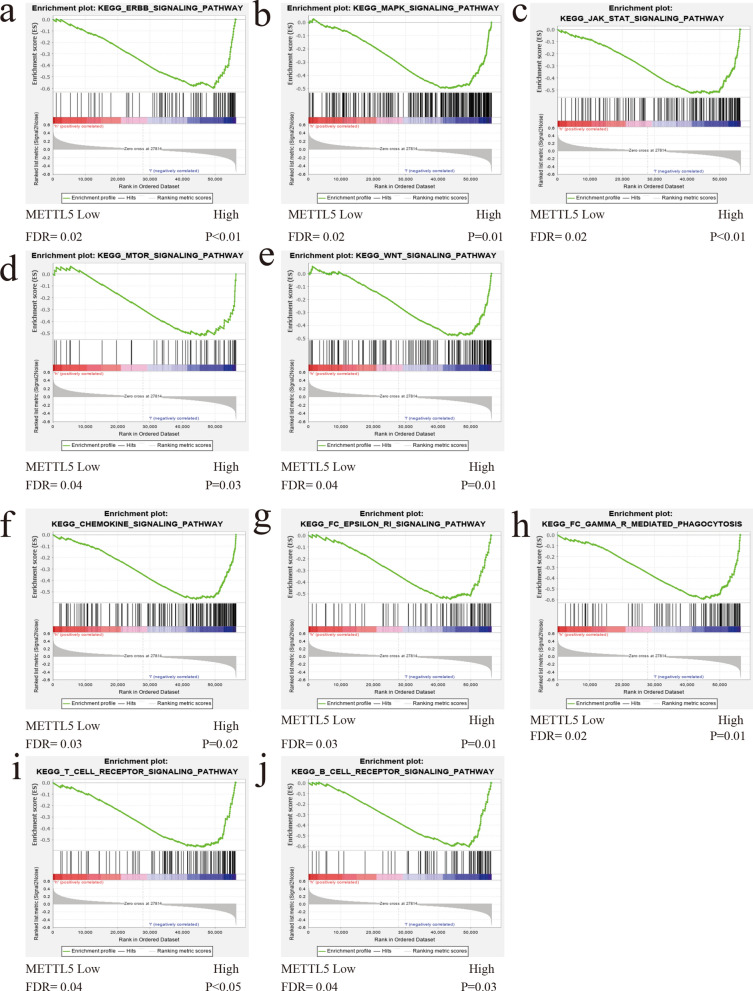
Results of enrichment analysis in GC of low-METTL5 expression groups

**Fig. 7:**
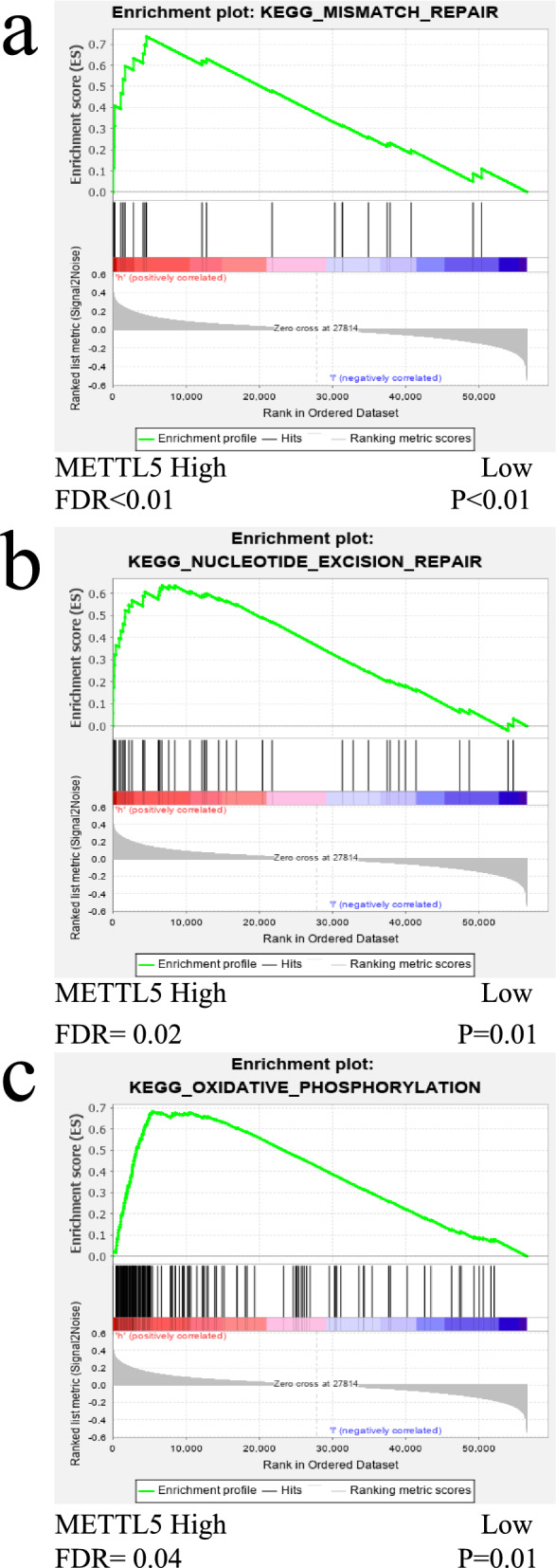
Results of enrichment analysis in GC of high-METTL5 expression groups

In order to determine the function and pathway of METTL5, GC data from TCGA were analyzed. GSEA was applied to enrich the functional pathways related to METTL5 based on the high- and low-groups. Afterwards, the genes of the low expression group of METTL5 were mainly enriched in some known key carcinogenic signaling pathways, including ERBB, MAPK, JAK-STAT, WNT, mTOR, and some immune-related pathways, including Fc-gamma R mediated phagocytosis, Fc-epsilon Ri, chemokine, T cell receptor and B cell receptor signaling pathway (Fig. 6, Table 4). While genes in the high expression group were mainly enriched in oxidative phosphorylation, nucleotide excision repair and mismatch repair (Fig. 7, Table 5).

**Table 4:** The KEGG of GSEA of the low-METTL5 expression groups

NAME	ES	NES	NOM p-val	FDR q-val
KEGG_ERBB_SIGNALING_PATHWAY	– 0.60	− 2.15	< 0.01	0.02
KEGG_MAPK_SIGNALING_PATHWAY	− 0.50	− 1.99	0.01	0.04
KEGG_FC_GAMMA_R_MEDIATED_PHAGOCYTOSIS	− 0.60	− 1.97	0.01	0.02
KEGG_JAK_STAT_SIGNALING_PATHWAY	− 0.53	− 1.93	< 0.01	0.02
KEGG_FC_EPSILON_RI_SIGNALING_PATHWAY	− 0.55	− 1.87	0.01	0.03
KEGG_CHEMOKINE_SIGNALING_PATHWAY	− 0.57	− 1.85	0.02	0.03
KEGG_WNT_SIGNALING_PATHWAY	− 0.48	− 1.79	0.01	0.04
KEGG_B_CELL_RECEPTOR_SIGNALING_PATHWAY	− 0.61	− 1.78	0.03	0.04
KEGG_MTOR_SIGNALING_PATHWAY	− 0.52	− 1.75	0.03	0.04
KEGG_T_CELL_RECEPTOR_SIGNALING_PATHWAY	− 0.56	− 1.74	< 0.05	0.04

**Table 5:** The KEGG of GSEA of the high-METTL5 expression groups

NAME	ES	NES	NOM p-val	FDR q-val
KEGG_OXIDATIVE_PHOSPHORYLATION	0.68	2.44	< 0.01	< 0.01
KEGG_NUCLEOTIDE_EXCISION_REPAIR	0.64	1.92	0.01	0.02
KEGG_MISMATCH_REPAIR	0.74	1.81	0.01	0.04

## Discussion

GC is a disease with high mortality and few effective diagnosis and treatment schemes. Therefore, it is urgent to find new biological markers for the diagnosis and prognosis of GC. METTL5 is a ribosomal RNA m^6^A methyltransferase, and its expression, function and prognosis in GC are still unclear. In our study, for the first time, we used immunohistochemistry to probe into the expression and prognostic role of METTL5 in GC, and our results suggested that METTL5 may serve as a new biomarker for diagnosis and prognosis evaluation of GC.

Firstly, the expression level of METTL5 in GC was significantly lower than that in adjacent tissues. Meanwhile, METTL5 was negatively correlated with clinicopathologic stage, suggesting that METTL5 may play a pivotal role in the progression of GC. It was reported that the expression METTL5 protein was increased in breast cancer specimens. Sun et al. [20] found that METTL5 protein was highly expressed in lung adenocarcinoma through bioinformatics analysis, which was different from what we found. We speculated that the expression characteristics of METTL5 protein in various organ tissues may be different. The similar phenomenon has been reported that the increased m^6^A methyltransferase METTL14 in breast cancer tissue promoted the migration and invasion of cancer cells, while METTL14 protein was down-regulated markedly in colorectal cancer to inhibit the proliferation and metastasis of colorectal cancer [21].

This study also investigated the effect of METTL5 protein expression on the overall survival rate of GC patients. Kaplan–Meier survival analysis and multivariate Cox survival analysis showed that METTL5 was an independent predictor of prognosis in patients with GC. Patients with high METTL5 expression group had better prognosis. Further, we constructed a nomogram to evaluate GC patients' 3-year and 5-year survival utilizing multivariate Cox analysis based on METTL5 expression and other four clinicopathological parameters. This model had a good predictive performance with satisfactory C-indexes (0.827), calibration curves and AUC values (0.852 for 3-years and 0.823 for 5-years), compared with several known prediction models for GC [22, 23]. We found that when the overall score was more than 105 points, the prognosis of GC patients was very poor, which should be paid attention to by doctors and patients and timely intervention measures should be taken. In addition, we also found that both data from TCGA database and GEO database suggested that GC patients with high METTL5 expression group had better prognosis, which may further validate the impact of METTL5 on the prognosis of GC patients. Therefore, it is necessary to pay attention to the effect of this protein on the prognosis of GC.

We further explored the possible mechanism of abnormal expression of METTL5 in GC. GSEA analysis showed that METTL5 low expression group was related to some oncogenic signaling pathways such as ERBB, MAPK, JAK-STAT, Wnt, and mTOR. All of these pathways have been reported to play a pivotal role in the occurrence and development of various cancers. For instance, inhibition of METTL14 promotes the proliferation and invasion of GC cells by activating Wnt pathway [24], downregulation of METTL3 promotes metastasis of colorectal cancer cells through MAPK pathway [25]. It also enriched some immune pathways including Fc-gamma R mediated phagocytosis, Fc-epsilon Ri, chemokine, T cell receptor and B cell receptor signaling pathway. Wang et al. [26] reported that RNA m^6^A modifications are strongly associated with innate immune response, T cells and the adaptive immune response. On the other hand, the related-pathways of high METTL5 group were oxidative phosphorylation, nucleotide excision repair and mismatch repair. It was reported that DNA base mismatch repair pathway was enriched in low-risk GC subtypes [27]. Above-mentioned results indicated that the role and mechanism of METTL5 in GC was associated with its expression status, which may be the reason why GC with various METTL5 expression was in different clinical stage and needs to be further explored.

## Conclusions

In conclusion, the protein expression of ribosomal RNA m^6^A methyltransferase METTL5 in GC was significantly decreased compared with adjacent tissues. Low expression of METTL5 protein was closely associated with poor prognosis of GC patients, and can serve as an independent predictor of prognosis of GC. A nomogram was constructed to evaluate GC patients’ prognosis based on METTL5 expression, which had a good predictive performance. Function of METTL5 in GC was associated with its expression status. METTL5 protein may work in the diagnosis and prognosis of GC, and it is expected to become a promising biomarker for GC.

## Supplementary Information

**Additional file 1: Table S1.** Summary of GEO datasets.

## Data Availability

The datasets analyzed during the current study are available in the TCGA repository (https://cancergenome.nih.gov/). The authors declare that the data supporting the fundings of this study are available within the article.

## Abbreviations

GC: Gastric cancer
GCTs: Gastric cancer tissues
ANTs: Adjacent normal tissues
AIMTs: Adjacent intestinal metaplasia tissues
C-index: Harrell's concordance index
ROCs: Receiver operating characteristics curve
AUC: The area under ROC curve
TCGA: Cancer Genome Atlas
GEO: Gene Expression Omnibus
IRS: Immunoreactivity Score
