# Supplementary material for: Expression and prognostic potential of ribosome 18S RNA m6A methyltransferase METTL5 in gastric cancer
Source: Cancer Cell Int. 2021 Oct 26;21:569. doi: 10.1186/s12935-021-02274-3 (PMC8549223; doi:10.1186/s12935-021-02274-3)
Supplement: Supplementary file 1 — Additional file 1: Table S1. Summary of GEO datasets. [file 12935_2021_2274_MOESM1_ESM.docx]

**Table S1: Summary of GEO datasets.**

| **GEO gene set ID** | **Platform** | **Experiment type** | **No. of samples** | **Organism** | **Overall design** | **PubMed ID** |
| --- | --- | --- | --- | --- | --- | --- |
| **GSE14210** | GPL571 [HG-U133A_2] Affymetrix Human Genome U133A 2.0 Array | Expression profiling by array | 167 | Homo sapiens | Endoscopic biopsy samples were collected from CF-treated metastatic gastric cancer patients prior to therapy and following the development of resistance to therapy. | 21364753/ 21173787 |
| **GSE15459** | GPL570 [HG-U133_Plus_2] Affymetrix Human Genome U133 Plus 2.0 Array | Expression profiling by array | 200 | Homo sapiens | Profiling of 200 primary gastric tumors on Affymetrix GeneChip Human Genome U133 Plus 2.0 Array. | 19798449/  21471434/  25008978/  25053715/  23684942/  30115935 |
| **GSE22377** | GPL570 [HG-U133_Plus_2] Affymetrix Human Genome U133 Plus 2.0 Array | Expression profiling by array | 43 | Homo sapiens | Tumor tissue samples were controlled for tumor contents higher than 75% and pathologically evaluated by two independent blinded pathologist. Total RNA was extracted and controlled for high quality (Agilent Bioanalyzer RIN above 7). | 21701537 |
| **GSE29272** | GPL96 [HG-U133A] Affymetrix Human Genome U133A Array | Expression profiling by array | 268 | Homo sapiens | cardia and non-cardia gastric tumors and normal glands | 23717493/ 24867265 |
| **GSE51105** | GPL570 [HG-U133_Plus_2] Affymetrix Human Genome U133 Plus 2.0 Array | Expression profiling by array | 94 | Homo sapiens | Profiling of 94 primary gastric tumors on Affymetrix GeneChip Human Genome U133 Plus 2.0 Arrays. | 24658156/ 29670108 |
| **GSE62254** | GPL570 [HG-U133_Plus_2] Affymetrix Human Genome U133 Plus 2.0 Array | Expression profiling by array | 300 | Homo sapiens | ACRG Gastric cohort: microarray profiles from 300 gastric tumors from gastric cancer patients. | 25894828/ 29725014 |
